# Bariatric Surgery Outcomes in Asians with Severe Obesity (BMI ≥ 50 kg/m^2^)

**DOI:** 10.3390/jcm15093305

**Published:** 2026-04-26

**Authors:** Sarah Ying Tse Tan, Trilene Ruiting Liang, Jasmine Kai Ling Chua, Hong Chang Tan, Emily Tse Lin Ho, Jean Paul Kovalik, Orlanda Qi Mei Goh, Chin Hong Lim, Alvin Kim Hock Eng, Weng Hoong Chan, Eugene Kee Wee Lim, Jeremy Tian Hui Tan, Angelina Xiangying Foo, Phong Ching Lee

**Affiliations:** 1 Department of Endocrinology, Singapore General Hospital, Singapore 169856, Singapore; 2 Department of Internal Medicine, Singapore General Hospital, Singapore 169608, Singapore; 3 Department of Upper Gastrointestinal & Bariatric Surgery, Singapore General Hospital, Singapore 168582, Singapore; 4 Department of Speciality Nursing, Singapore General Hospital, Singapore 169608, Singapore

**Keywords:** severe obesity, high BMI, metabolic bariatric surgery, sleeve gastrectomy, gastric bypass

## Abstract

**Background:** The benefits of metabolic bariatric surgery (MBS) in people with a body mass index (BMI) ≥ 50 kg/m^2^ are not well-established, with concerns of increased risk and poorer weight loss. The optimal surgical type (gastric bypass [GB] versus sleeve gastrectomy [SG]) is unclear, with studies comparing complication rates, weight loss, and glycaemic outcomes reporting mixed results. **Methods:** Participants with a BMI ≥ 50 kg/m^2^ undergoing MBS (SG or GB) from 2008 to 2022 were recruited. Demographics, anthropometrics, biochemistry, and diabetes status were analysed at baseline, 12 months, and 24 months post-operatively. Surgical outcomes and complications were analysed. **Results:** The study included *n* = 184, with BMI ≥ 50 kg/m^2^ (57.6% female, age 38.6 ± 10.5 years, and BMI 55.3 ± 6.0 kg/m^2^). Pre-operatively, 21.1% had pre-diabetes, and 33.2% had diabetes (mean HbA1c 8.0 ± 1.7%). Most subjects (89.1%) underwent SG. The overall 30-day adverse event rate was 4.9%, with a higher, but not statistically significant, rate in the GB group (15.0% vs 3.7%, *p* = 0.061). The GB group had a longer length of stay (GB =4.5 ± 0.6 days, SG = 3.1 ± 0.2, and *p* = 0.023). The rate of revisional surgery was 2.7%, with no significant difference between groups. The follow-up rate was 67.9% at 12 months and 51.1% at 24 months. The average %total weight loss (%TWL) at 12 months (27.4 ± 9.0%, SG = 27.6 ± 9.0%, GB = 26.0 ± 9.4%, and *p* = 0.481) and 24 months (27.1 ± 10.9%, SG = 27.4 ± 11.1%, GB = 24.9 ± 8.9%, and *p* = 0.495) were similar between groups. The GB group had a larger HbA1c reduction (3.2 ± 1.1%) than SG (1.9 ±1.3%, *p* = 0.030) but no difference in diabetes remission rates (69.2% at 12 months, 76.7% at 24 months). **Conclusions:** MBS is safe and effective for individuals with a BMI ≥ 50 kg/m^2^, with low complication rates and good weight loss and glycaemic outcomes at 2 years. No statistically significant differences in %TWL, diabetes remission, or complication rates were noted between SG and GB groups, though results are limited by the small number of participants who underwent GB.

## 1. Introduction

Metabolic bariatric surgery (MBS) is an effective treatment option for people with clinically severe obesity and has been shown to produce significant and durable long-term weight loss, improvement and resolution of obesity-related complications, and reduction in mortality [1,2,3]. The two most commonly performed procedures, the sleeve gastrectomy (SG) and Roux-en-Y gastric bypass (RYGB), are generally safe, with perioperative mortality rates ranging from 0.03 to 0.2%, and 30-day risks of serious adverse events of <6% [4].

However, the benefits and risks of MBS in people with severe obesity with a body mass index (BMI) ≥ 50 kg/m^2^ are not as well-established [5]. There is a higher burden of obesity-related complications such as diabetes and hypertension [6], potentially increasing the operative and anaesthetic risks in this population. Surgical anatomy may also be more challenging due to the higher amount of visceral adiposity, which could result in longer operating times and the rate of surgical complications. Existing data on surgical complications post-MBS in people with a BMI ≥ 50–60 kg/m^2^ is mixed, with some studies reporting an increase in post-operative complications, 30-day adverse events, and mortality [6,7,8,9], and others reporting no difference in adverse outcomes [10,11,12]. Weight loss outcomes post-MBS in this population are also a concern, as a high pre-operative BMI is traditionally associated with less post-operative weight loss [13]. Lastly, there is no consensus on the recommended type of surgery in this population, with some studies advocating for RYGB due to potentially greater long-term weight loss [7,14], while others opt for SG, citing a lower rate of surgical complications [9].

Studies from Asia on MBS for people with a very high BMI (≥50 kg/m^2^) are scarce. At a given BMI, Asians have higher visceral adiposity than White people [15], potentially increasing the technical difficulty of MBS in this population. Asians are also described to have inferior weight loss outcomes post-MBS compared to White people [16]. Thus, the risks and benefits of MBS amongst Asian people with a very high BMI (≥50 kg/m^2^) require further assessment.

This study aimed to evaluate the outcomes of MBS in Asian people with a very high BMI (≥50 kg/m^2^), including post-operative complications, mortality, weight loss outcomes, and diabetes remission. As an exploratory aim, outcomes were also compared between people who underwent SG versus gastric bypass (GB).

## 2. Methods

### 2.1. Participants

This retrospective study was conducted at a tertiary centre. All consecutive patients with BMI ≥ 50 kg/m^2^ who underwent metabolic bariatric surgery (MBS) between January 2008 and December 2022 were screened for eligibility. A total of 184 patients met the study criteria and were included in the final analysis. Inclusion criteria were (1) pre-operative BMI ≥ 50 kg/m^2^, and (2) completion of either sleeve gastrectomy (SG) or gastric bypass (GB). Exclusion criteria included (1) prior bariatric surgery, (2) incomplete baseline data, and (3) lack of any post-operative follow-up data. All bariatric procedures were performed by our team of experienced bariatric surgeons, using techniques that we have reported previously for SG, RYGB, and one-anastomosis gastric bypass (OAGB) [17,18]. Patients were managed by a multidisciplinary team including bariatric surgeons, endocrinologists, dietitians, and physiotherapists pre- and post-operatively. A very low caloric diet (VLCD) was prescribed for all patients for 2 weeks pre-operation as part of routine clinical care.The study was approved by the institution’s Centralised Institutional Review Board, and waiver of signed informed consent was granted.

Baseline demographic and anthropometric data were collected. Post-operative outcomes including operation time, length of stay, rate of 30-day adverse events (including surgical complications, readmissions, and re-operations), rate of revisional surgery, and mortality were recorded. Weight and glycaemic outcomes (including diabetes remission, HbA1c, and diabetes medications) were measured pre-operatively, 12 months post-operatively, and 24 months post-operatively. Post-operative weight loss was measured by percentage of total weight loss (%TWL). Diabetes remission was defined as HbA1c < 6.5% without the use of diabetes medications for 3 months [19].

### 2.2. Statistical Analysis

Statistical analysis was performed using IBM SPSS Statistics 26.0 (Armonk, NY, USA: IBM Corp). Descriptive statistics were computed and expressed as mean ± standard deviation (continuous variables) and frequency (categorical variables). Post-operative outcomes amongst the SG group and RYGB group were compared using Student’s *t*-tests and Pearson’s χ^2^ tests for continuous and categorical variables, respectively. *p* < 0.05 were regarded to indicate nominal statistical significance.

## 3. Results

### 3.1. Baseline Data

A total of 184 participants were included in this study, of whom 164 (89.1%) underwent SG and 20 (10.9%) underwent gastric bypass (GB). At baseline, the mean age was 38.6 ± 10.5 years old, and 57.6% were female. Ethnic distribution included 17.9% Chinese, 50.5% Malay, 23.4% Indian, and 8.2% others (including White and Eurasian). The mean pre-operative weight was 148.23 ± 22.0 kg, and the mean pre-operative BMI was 55.3 ± 6.0 kg/m^2^. The prevalence of obesity-related complications was high: 39 (21.2%) had pre-diabetes, 61 (33.2%) had diabetes, and 86 (46.7%) had pre-hypertension or hypertension. Of those with diabetes, the mean pre-operative HbA1c was 8.0 ± 1.7%. There was no significant difference in pre-operative weight or BMI amongst participants who underwent SG and GB. However, significantly more participants who underwent GB had obesity-related complications compared to those who underwent SG, including diabetes or pre-diabetes, hypertension, and hyperlipidaemia. Baseline characteristics are described in Table 1.

### 3.2. Post-Operative Outcomes

The GB group had a significantly longer mean operating time (GB = 249.4 ± 76.3 min, SG = 112.4 ± 43.4 min, and *p* < 0.0001) and length of stay (GB = 4.5 ± 0.6 days, SG = 3.1 ± 0.2, and *p* = 0.023) compared to the SG group. The overall rate of 30-day adverse events was 4.9% (*n* = 9), including haemorrhage (*n* = 3), infection (*n* = 3), leak (*n* = 1), venous thrombosis (*n* = 1), and ureteric stone (*n* = 1). The rate of 30-day adverse events was higher in the GB group (15%) compared to the SG group (3.7%), with a trend towards but not meeting statistical significance (*p* = 0.061). The rate of revisional surgery was 2.7%, with no significant difference between SG and GB groups. Median time from primary procedure to revisional surgery was 79.8 months (32 to 128 months). There were no 30-day mortalities in this cohort. Post-operative outcomes are summarised in Table 2.

### 3.3. Weight Outcomes

**Figure 1:**
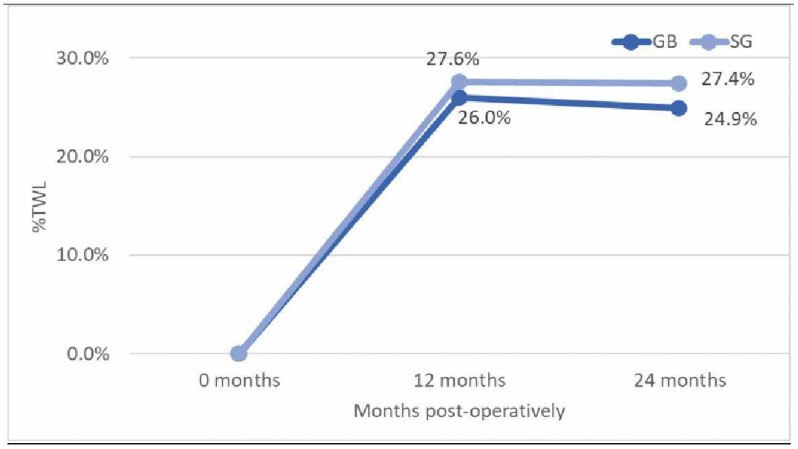
Percentage of total weight loss at 12 and 24 months post-operatively. No significant difference in %TWL between SG and GB groups at 12 months (*p* = 0.481) and 24 months (*p* = 0.495).

Overall, participants had a %TWL of 27.4 ± 9.0% at 12 months and 27.1 ± 10.9% at 24 months. There was no significant difference in %TWL between the SG and GB groups at 12 months (SG = 27.6 ± 9.0%, GB = 26.0 ± 9.4%, and *p* = 0.481) and 24 months (SG = 27.4 ± 11.1%, GB = 24.9 ± 8.9%, and *p* = 0.495) post-operatively. Weight outcomes are summarised in Table 3 and represented in Figure 1.

Follow-up rate was 67.9% at 12 months and 51.1% at 24 months, with no difference in follow-up rates between the SG and GB groups at 24 months (SG = 51.2%, GB = 50%, and *p* = 0.553). There was no significant difference in the baseline characteristics of participants with and without follow-up data at 24 months.

### 3.4. Glycaemic Outcomes

Amongst participants with pre-existing DM (*n* = 61), the mean HbA1c was significantly lower post-operatively at 12 months (5.5 ± 0.5%, *p* < 0.001) and 24 months (5.7 ± 0.8%, *p* < 0.001) compared to the baseline (8.0 ± 1.7%). The rate of remission of diabetes was 69.2% at 12 months and 76.7% at 24 months, with no statistically significant difference between the SG and GB groups. There was no statistically significant difference in the mean HbA1c in the SG and GB groups at 12 months (SG = 5.6 ± 0.5%, GB = 5.3 ± 0.3%, and *p* = 0.186) and 24 months (SG = 5.7 ± 0.7%, GB = 5.7 ± 0.9%, and *p* = 0.934) post-operatively. However, the GB group had a larger HbA1c reduction at 24 months (3.2 ± 1.1%) compared to the SG group (1.9 ± 1.3%, *p* = 0.030). Glycaemic outcomes are summarised in Table 4 and Table 5.

## 4. Discussion

This study showed that MBS in people with a BMI ≥ 50 kg/m^2^ produces good weight loss and glycaemic outcomes at 12 and 24 months post-operatively and is generally safe with a low rate of complications. There was no difference in weight outcomes at 12 and 24 months between the groups that underwent SG and GB. The overall diabetes remission rate was good (76.7%) at 24 months post-operatively, with no significant difference between the SG and GB groups. However, participants with diabetes who underwent GB had a significantly greater HbA1c reduction at 24 months compared to those who underwent SG. Those who underwent GB had a higher rate of 30-day adverse events compared to the SG group, although this was not statistically significant.

The treatment of people with severe obesity, traditionally defined as having a BMI ≥ 50–60 kg/m^2^, is challenging. The burden of obesity-related complications increases as the BMI increases [6], with higher rates of multiple complex comorbidities. In this study, more than half the cohort had diabetes or pre-diabetes (54.4%). Amongst those with diabetes, control was poor, with a mean HbA1c of 8.0 ± 1.7%.

In light of the high BMI and multiple obesity-related complications, lifestyle therapy and even novel pharmacotherapy agents are unlikely to produce adequate clinically significant weight loss in this population. MBS would be the treatment of choice, as it is able to produce greater and more durable weight loss as well as improvement in obesity-related complications compared to pharmacological options [1]. However, people with a high BMI are more likely to have greater levels of visceral adiposity and more medical comorbidities, which can lead to increased surgical and anaesthetic risks [20]. In this study, the rate of post-operative 30-day adverse events was reassuringly low at 4.9%, in keeping with rates reported in general cohorts undergoing MBS [4,21]. A study by Howell looking at 208 people with a BMI ≥ 60 kg/m^2^ undergoing RYGB or SG also reported a similar complication rate of 5.3% [22]. Several other studies comparing the rate of early complications and mortality post-MBS showed no difference between people with a BMI < 60 kg/m^2^ and those with a BMI ≥ 60 kg/m^2^ [10,11,23]. However, most experts advocate that MBS on patients with a BMI over 50 kg/m^2^ should be performed by experienced bariatric surgeons with the support of bariatric anaesthesiologists [24].

Participants who underwent GB had a higher rate of 30-day adverse events (15%) compared to those who underwent SG (3.7%), although this was not statistically significant. Similar findings were reported in a study by Wilkinson et al., a database study that analysed the outcomes of 24,940 people with a BMI of 50–60 kg/m^2^ and 5723 people with a BMI > 60 kg/m^2^ who underwent RYGB and SG [9]. People with a BMI > 60 kg/m^2^ who underwent RYGB had a higher rate of post-operative complications (including unplanned intubations, intensive care unit admissions, blood transfusions, readmissions, and re-operations) compared to those who underwent SG [9].

The trend towards a higher rate of post-operative complications in patients undergoing RYGB must be weighed against long-term weight and metabolic outcomes, which have traditionally been described to be superior compared to SG [25]. Weight loss and metabolic outcomes post-MBS are not as clear in people with a BMI ≥ 50 kg/m^2^, especially since a high pre-operative BMI is associated with lower post-operative weight loss. In this study, good weight loss outcomes of 27–28% TWL on average were achieved at 12 and 24 months, with no significant difference between the SG and GB groups. Results are similar to the %TWL achieved by our general population undergoing MBS (previously reported) [13]. However, longer-term studies may be needed to compare the durability of weight loss between GB and SG. There are several studies looking at mid- to long-term weight loss outcomes post-MBS in patients with a BMI above 50–60 kg/m^2^, but results are mixed. Studies by Singla et al. and Gonzalez-Heredia et al. described greater weight loss in GB groups compared to SG groups at 12–24 months post-operatively [14,26]. On the other hand, Arapis et al. and Hong et al. assessed longer-term weight loss at 36–72 months post-MBS and described weight loss outcomes as being similar between the GB and SG groups [7,27].

Results on metabolic outcomes between the SG and GB groups are also mixed. On the whole, this study showed that MBS in patients with a BMI ≥ 50 kg/m^2^ yielded good rates of diabetes remission of 69.2% at 12 months and 76.7% at 24 months. Diabetes remission rates were higher than those previously reported in our general MBS cohort (55.9% at 12 months) [2]. A higher baseline BMI has been described as being a predictive factor for post-operative diabetes remission [28].

In this study, there was no difference in remission rates between the GB and SG groups. However, participants with diabetes who underwent GB did have a significantly greater HbA1c reduction (3.2 ± 1.1%) at 24 months compared to the SG group (1.9 ± 1.3%). This difference was not appreciated at 12 months post-operatively, suggesting that the metabolic benefits of GB over SG may be more apparent and durable in the long run. Most other studies described comparable diabetes remission rates between SG and GB, with the duration of follow-up ranging from 12 to 41 months post-MBS [26,27,29]. However, two studies showed superior diabetes remission rates with GB compared to SG, with one study by Thereaux et al. describing a diabetes remission rate of 70.7% amongst GB patients compared to 47.5% amongst SG patients at 1 year post-MBS [7,30]. Notably, none of these studies reported the change in HbA1c level post-operatively.

This is one of the few studies to assess the MBS outcomes in people with a very high BMI in an Asian population. Compared to White people, Asians have a higher amount of visceral adiposity and higher risk of developing diabetes at a given BMI [31]. This potentially poses increased surgical and anaesthetic risks, especially amongst Asians with a BMI ≥ 50 kg/m^2^. These risks must be weighed against the benefits of MBS. However, weight loss outcomes are also a concern in Asians with a BMI ≥ 50 kg/m^2^, as a high pre-operative BMI and Asian ethnicity have both been described as being associated with poorer weight loss post-MBS. Two small studies in Asia (India and Singapore) described the overall safety of MBS in patients with a BMI > 47.5–50 kg/m^2^, with low rates of short-term complications [26,32]. Only short-term weight loss outcomes were reported (6 months and 1-year post-MBS, respectively). Post-operative weight loss at 6 months was not inferior compared to patients with a BMI < 47.5 kg/m^2^ in one study [32]. The other study compared SG and one-anastomosis GB (OAGB) outcomes in patients with a BMI ≥ 50 kg/m^2^ and described superior TWL in the OAGB group at 1 year post-operation (%TWL 39.9% versus 30%, *p* < 0.0001), but with similar rates of diabetes and hypertension remission. Our study adds to the body of evidence to demonstrate the safety and effectiveness of MBS in Asians with a BMI ≥ 50 kg/m^2^. This study also provides longer term weight and glycaemic outcome data and illustrated no difference in weight and diabetes remission between the SG and GB groups at 24 months post-MBS. However, the GB cohort did have greater HbA1c reduction compared to the SG cohort at 24 months.

This study has a few limitations. Firstly, the choice of bariatric procedure was not randomised, and substantially fewer patients underwent GB compared to SG. Those who underwent GB had a significantly higher rate of obesity-related complications, which may have confounded comparisons between groups, as differences in outcomes (including weight loss, glycaemic control, and complications) may be partly attributable to underlying patient risk profiles rather than the surgical procedure itself. Nonetheless, this could indicate intrinsic bias from the bariatric team and/or patients to opt for SG in this more complex group and is reflective of real-world practice. Follow-up rates were suboptimal (67.9% at 12 months, 51.1% at 24 months). Given the small number of participants with complete follow-up data, especially in the GB group, adjusted analyses such as multivariable regression or longitudinal mixed-effects modelling were not performed; therefore, comparisons between surgical groups should be interpreted as exploratory and unadjusted. This high rate of patient attrition post-MBS has been well described. Two studies reporting mid- to long-term bariatric outcomes in patients with a high BMI illustrated a dramatic decline in follow-up data after 12 months [7,27]. Importantly, there was no difference in follow-up rates between the SG and GB groups in this study. The rate of follow-up in this cohort was also higher than that of our general bariatric cohort (40.3% at 18 months) [13]. Lastly, follow-up data was only obtained up to two years post-operation, limiting any long-term conclusions.

Future studies exploring the role of pre-operative weight loss via bridging strategies such as a longer duration of pre-operative VLCD or the use of pharmacotherapy such as GLP-1RA-based therapies would be a useful area of research [24,33]. Longer-term follow-up data beyond 24 months would also provide greater insight into the durability of weight loss and metabolic benefits in people with a high BMI and clarify whether differences in outcomes between sleeve gastrectomy and gastric bypass emerge over time.

## 5. Conclusions

MBS is an effective and safe treatment option for severe obesity in Asians with a BMI ≥ 50 kg/m^2^, with successful weight loss outcomes and comorbidity resolution at 24 months post-operation.

In terms of choosing the bariatric procedure, no statistically significant differences were observed in weight loss or diabetes remission. However, comparisons between GB and SG in this study were exploratory in nature, and these findings should be interpreted with caution given the limited sample size and imbalance between groups. This study showed a trend towards increased risk in 30-day adverse events amongst patients who underwent GB; statistical significance was not achieved. Similarly, while other studies report mixed results, the current evidence remains inconclusive rather than demonstrating a clear benefit of one procedure over the other. The optimal bariatric procedure in this population remains an ongoing debate [34], although there is emerging expert opinion advocating for SG to be selected as a primary procedure, reserving RYGB as a revision procedure if weight loss is suboptimal [7,24]. Recent studies also explored the potential role of the duodenal switch procedure in this cohort, which demonstrated greater weight loss than RYGB, but with more adverse events [35,36]. Ultimately, management should be individualised, and larger, well-designed studies with longer follow-up are required to better compare the risks and long-term outcomes of different bariatric procedures in patients with a very high BMI.

## Figures and Tables

**Table 1:** Baseline characteristics.

Characteristics			Total (*n* = 184)	SG (*n* = 164)	GB (*n* = 20)	*p*-Value
Age (years)			38.6 ± 10.5	38.7 ± 10.5	37.9 ± 10.2	0.731
Sex		Male	78 (42.4%)	71 (43.3%)	7 (35%)	0.323
		Female	106 (57.6%)	93 (56.7%)	13 (65%)	
Race		Chinese	33 (17.9%)	29 (17.7%)	4 (20%)	0.896
		Malay	93 (50.5%)	81 (49.4%)	12 (60%)	
		Indian	43 (23.4%)	40 (24.4%)	3 (15%)	
		Others	14 (8.2%)	14 (8.5%)	1 (5%)	
Pre-operative weight (kg)			149.1 ± 21.5	149.7 ± 21.8	144.5 ± 18.7	0.105
Pre-operative BMI (kg/m^2^)			55.1 ± 7.3	56.2 ± 5.5	56.1 ± 6.6	0.104
Glycaemic status	No diabetes		84 (45.7%)	79 (48.2%)	5 (25%)	0.040
	Pre-diabetes		39 (21.2%)	35 (21.3%)	4 (20%)	
	Diabetes		61 (33.2%)	50 (30.5%)	11 (55%)	
Mean HbA1c of those with diabetes (%)			8.0 ± 1.7	7.9 ± 1.8	8.2 ± 1.2	0.576
Pre-hypertension or hypertension			86 (46.7%)	72 (43.9%)	14 (70%)	0.024
Hyperlipidaemia			44 (23.9%)	34 (20.7%)	10 (50%)	0.007

**Table 2:** Post-operative outcomes.

Outcomes		Total (*n* = 184)	SG (*n* = 164)	GB (*n* = 20)	*p*-Value
Operating time (minutes) *		125.6 ± 62.3	112.4 ± 43.4	249.4 ± 76.3	<0.0001
Length of stay (days)		3.3 ± 2.5	3.1 ± 0.2	4.5 ± 0.6	0.023
30-day adverse events (%)		9 (4.9%)	6 (3.7%)	3 (15%)	0.061
Types of adverse events	Haemorrhage	3	3	0	-
	Infection	3	1	2	
	Leak	1	0	1	
	Venous thrombosis	1	0	1	
	Ureteric stone	1	0	1	
Revisional surgery (%)		5 (2.7%)	4 (2.4%)	1 (5%)	0.441
30-day mortality (%)		0	0	0	-

* Excluding those who underwent concurrent operations (*n* = 18).

**Table 3:** Post-operative weight loss.

Outcomes	Total	SG	GB	Mean Difference (95% Confidence Interval)	*p*-Value
% TWL at 12 months (*n* = 125; SG = 107; GB = 18)	27.4 ± 9.0	27.6 ± 9.0 (0.9)	26.0 ± 9.4 (2.2)	2.3 (−2.9–6.1)	0.481
% TWL at 24 months (*n* = 94; SG = 84; GB = 10)	27.1 ± 10.9	27.4 ± 11.1 (1.2)	24.9 ± 8.9 (2.8)	3.7 (−4.8–9.8)	0.495

Data is presented as mean ± standard deviation (standard error mean).

**Table 4:** HbA1c at 12 and 24 months for patients with diabetes.

Outcomes	Total	SG	GB	*p*-Value
HbA1c at 12 months (%)	5.5 ± 0.5	5.6 ± 0.5	5.3 ± 0.3	0.186
HbA1c reduction at 12 months (%)	2.4 ± 1.7	2.1 ± 1.8	3.3 ± 1.9	0.017
HbA1c at 24 months (%)	5.7 ± 0.8	5.7 ± 0.7	5.7 ± 0.9	0.934
HbA1c reduction at 24 months (%)	2.2 ± 1.4	1.9 ± 1.3	3.2 ± 1.1	0.030

**Table 5:** Post-operative diabetes remission rates.

Outcomes	Total	SG	GB	*p*-Value
Diabetes remission rate at 12 months (*n* = 39)	27 (69.2%)	21 (67.7%)	6 (75%)	0.527
Diabetes remission rate at 24 months (*n* = 30)	23 (76.7%)	18 (78.3%)	5 (71.4%)	0.532

## Data Availability

Data is available from the corresponding author upon reasonable request.
